# Performance evaluation of Siemens Atellica enhanced estradiol assay

**DOI:** 10.1515/almed-2019-0009

**Published:** 2020-01-07

**Authors:** Laura Macias-Muñoz, Xavier Filella, Josep Maria Augé, Felicia A. Hanzu, Manuel Morales-Ruiz, Josep Lluis Bedini, Wladimiro Jiménez, Gregori Casals

**Affiliations:** Department of Biochemistry and Molecular Genetics, Hospital Clínic, IDIBAPS, Barcelona, Spain; Department of Endocrinology, Hospital Clínic, IDIBAPS, Barcelona, Spain; University of Barcelona, Barcelona, Spain; Department of Biochemistry and Molecular Genetics, Hospital Clinic Universitari, Villarroel 170, Barcelona 08036, Spain

**Keywords:** estradiol, immunoassay, method comparison, performance evaluation

## Abstract

**Background:**

Serum estradiol (E2) levels may be used in the diagnostic and/or monitoring of a broad variety of clinical conditions. The aims of this study were to evaluate the Siemens enhanced estradiol assay (eE2) on Atellica IM 1600 (Siemens Healthineers) and to perform a sample comparison with the Siemens ADVIA Centaur XP (Siemens Healthineers).

**Methods:**

Within-day and between-day coefficient of variation (CV) were determined using serum sample pools and quality control materials. Six serum samples with decreasing concentrations of E2 were used to assess the limit of quantification. Linearity was evaluated using two different serum samples. Accuracy was evaluated by measuring three certified reference materials. Passing–Bablok regression and Bland–Altman plot were used for comparing Atellica and Centaur XP in 119 serum samples ranging from 45 to 10,059 pmol/L.

**Results:**

Within-day and between-day imprecision was <6.6%. Accuracy was <6.0% for all values except for 114 pmol/L (22%). The study of limit of quantification resulted in an interday imprecision <20%. High correlation between measured and expected estradiol dilution results was observed (*R* = 0.99), with recoveries ranging from 77 to 93%. Comparison study showed good agreement and no significant bias.

**Conclusions:**

The study shows that Atellica eE2 assay presents acceptable imprecision and accuracy and correlates well with Centaur XP.

## Introduction

Estradiol (17β-estradiol) (E2) is the most potent one of naturally occurring estrogens and also the most widely analyzed one in clinical routine and research laboratories. The determination of this C18-steroid hormone in serum provides relevant information in many endocrinological or gynecological conditions. Thus, E2 measurements are useful in assisted reproduction to monitor follicular development and in the clinical evaluation of hypogonadism, hirsutism, polycystic ovary syndrome, amenorrhea, female infertility, menopause, gonadal tumors, and feminization in males or monitoring of aromatase inhibitor therapy in breast cancer, among others [1]. Physiological levels of E2 are low (often <184 pmol/L) in the serum of men, children, and postmenopausal women, but they can range widely (110–2,937 pmol/L) in premenopausal women [2]. These differences in the expected levels of serum E2 are even larger, spanning across five orders of magnitude, when considering the variety of its clinical applications. Thus, for example, serum E2 concentration may reach up to 36,713 pmol/L during testing performed in support of in vitro fertilization programs, whereas aromatase inhibitor therapy can decrease serum E2 concentration to 3.7 pmol/L [3]. In summary, serum E2 presents a wide range of reference intervals and clinical applications that determine a need to maintain analytical quality specifications in a wide analytical range. In this regard, assessment of the analytical capacity of an E2 assay is critical when considering its reliability to answer specific clinical or research questions [2].

High-throughput automated direct (unextracted) immunoassays allow the growing clinical need for E2 quantitation and are convenient to be implemented by most hospital clinical laboratories. However, awareness of loss of analytical specificity and accuracy at low concentrations of direct immunoassays has grown over the last few years [4], [5], [6], [7], [8], [9], [10], [11]. Very recently, two new analyzers (Atellica IM 1300 and Atellica IM 1600, Siemens Healthineers, Erlangen, Germany) are incorporated and expected to expand into many clinical laboratories. In this regard, we evaluated the analytical performance of the direct immunoassay Atellica IM 1600 enhanced estradiol assay (eE2) (Siemens Healthineers, Erlangen, Germany). Particularly, we focused our attention on the assessment of imprecision, accuracy, limit of quantification, assay linearity, and method comparison while paying special attention to eE2 low analytical range.

## Materials and methods

### Assay principle and instrument

The Atellica IM eE2 assay is a competitive immunoassay by chemiluminometric technology based on a sheep monoclonal antiestradiol antibody labeled with acridinium ester. First, the endogenous E2 present in the serum sample is released from its binding proteins by an ancillary releasing agent. Then, the labeled antibody is added to bind available E2. An E2 capture conjugate coupled to magnetic latex particles is also added to the reaction, and it competes with the endogenous E2 for the binding of the labeled antibody. After washing, the dispensation of acid and base initiates the chemiluminescent reaction. An inverse relationship exists between the amount of E2 present in the serum sample and the amount of relative light units (RLUs) detected by the instrument. Measurements were performed in an Atellica IM 1600 analyzer. Time to first result is 18 minutes, with successive results in 20-second increments. The sample volume required is 80 μL. The detection limit and the limit of quantification provided by the manufacturer are 43 pmol/L and 70 pmol/L, respectively. The measuring interval ranges from 43 pmol/L to 11,014 pmol/L.

### Performance characteristics

#### Imprecision and accuracy

Intraday assay imprecision was assessed by testing aliquots of two serum pools, three levels of commercially available quality control materials (Liquichek Immunoassay Plus, Bio-Rad, Irvine, CA) and three certified reference materials (BCR-576, BCR-577, and BCR-578, Joint Research Center, European Commission). Five replicates of each sample were performed on a single day. Interday imprecision was calculated using the same samples on five different days. Certified reference materials BCR-576, BCR-577, and BCR-578 were also used to evaluate analytical intraday and interday accuracy of the assay. The acceptance criteria established for imprecision and accuracy studies were based on the desirable specifications derived from biological variation [12].

#### Limit of quantification

Limit of quantification was evaluated by testing six panels of serum pools with E2 average concentrations ranging between 52.9 and 223 pmol/L that were measured eight times on five different days. There is no current consensus on the required imprecision for clinical E2 measurements. We adopted a 20% coefficient of variation (CV) to define the limit of quantification as a value broadly used for this purpose [3], [13].

#### Assay linearity

Linearity was evaluated using two serums with different concentrations of E2 (1,957 and 3,748 pmol/L). Serial dilutions to obtain 1/2, 1/5, and 1/10 of the original concentration were performed for each sample with the specific assay diluent (Atellica IM eE2 DIL ReadyPack ancillary reagent).

#### Method comparison

One hundred nineteen serum specimens in the range of 44 to 10,059 pmol/L consecutively submitted to the clinical laboratory for E2 routine analysis were analyzed using Atellica IM 1600 and ADVIA Centaur XP (Siemens Healthineers) on the same day.

In addition, 29 serum samples that presented low E2 concentrations measured by the estradiol-sensitive ELISA EIA-4,399 (DRG International, Inc., Springfield, USA) (n = 4 samples <11 pmol/L, n = 16 between 11 and 43 pmol/L, n = 5 between 43 and 73 pmol/L, and n = 4 between 73 and 110 pmol/L) were also analyzed using the Atellica IM 1600. This is also a direct immunoassay but with a long period of incubation of 4 h and a short analytical range of 0–734 pmol/L that allows lower limit of detection (LOD) according to the manufacturer (5.1 pmol/L).

### Statistical analysis

Statistical analysis was carried out using Microsoft Excel and RStudio (version 1.1.463 – © 2009–2018 RStudio, Inc.). Imprecision at each concentration level and limit of quantification were expressed as CV. Accuracy was determined as the difference between measured E2 concentrations with nominal concentrations expressed in percent. Linearity of serial dilutions at different E2 levels was determined by linear regression analysis, and recovery was calculated as follows: [(measured − expected)/expected] × 100. Linearity was assumed when the correlation coefficient was >0.95. Passing–Bablok regression analysis was used to evaluate method comparisons, and difference plots (Bland–Altman plots) were constructed to assess systematic bias between assays.

This investigation has complied with the World Medical Association Declaration of Helsinki regarding ethical conduct of research involving human subjects.

## Results

### Imprecision and accuracy

Table 1 summarizes the values obtained in the imprecision study. Intraday and interday imprecision were observed to be <5.0% and <7.0%, respectively, at all the evaluated E2 concentrations (ranging from 175 to 3,848 pmol/L). Table 2 shows the values of accuracy and imprecision of three certified reference materials. Imprecision was <6.0% at all evaluated concentrations. Accuracy was <7.0% at medium (690 pmol/L) and high (1,340 pmol/L) E2 levels and was 22% (interday accuracy) at low E2 concentrations (114 pmol/L).

**Table 1:** Imprecision of estradiol measurements expressed as coefficient of variation.

Type of sample	Mean	Imprecision, %	
	Estradiol, pmol/L	Intraday, n = 5	Interday, n = 5
QC-1	175	4.0	6.5
QC-2	1,336	1.8	4.1
QC-3	3,848	1.2	1.0
Sample pool 1	903	3.5	6.6
Sample pool 2	3,301	2.4	2.2

qc, quality control; n, number of measurements.

**Table 2:** Imprecision and accuracy values of three certified reference materials.

Reference material	Target	Imprecision, %		Accuracy, %	
	Estradiol, pmol/L	Intraday, n = 5	Interday, n = 5	Intraday, n = 5	Interday, n = 5
BCR-576	114	5.9	4.6	29	22
BCR-577	690	1.5	1.8	0.7	0.0
BCR-578	1,340	1.4	2.1	6.9	5.2

n, number of measurements.

### Limit of quantification

**Figure 1:**
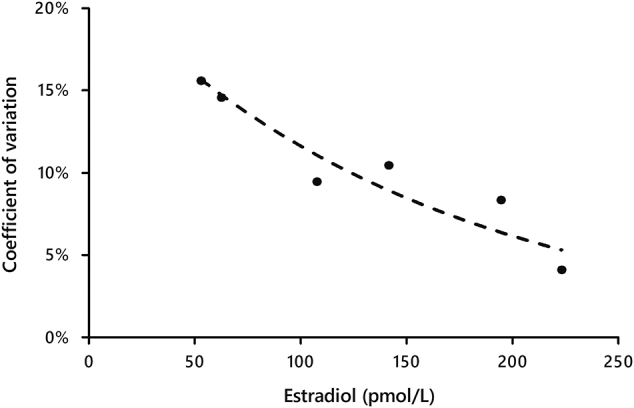
Limit of quantification of Atellica IM 1600 eE2 assay.

The imprecision of the assay was also assessed with decreasing concentrations of E2 using serum samples. All serum samples presented an interday imprecision <20%, 53 pmol/L being the lowest concentration evaluated. Minimum concentration of E2 corresponding to CV <10% was estimated to be 125 pmol/L (Figure 1).

### Assay linearity

**Figure 2:**
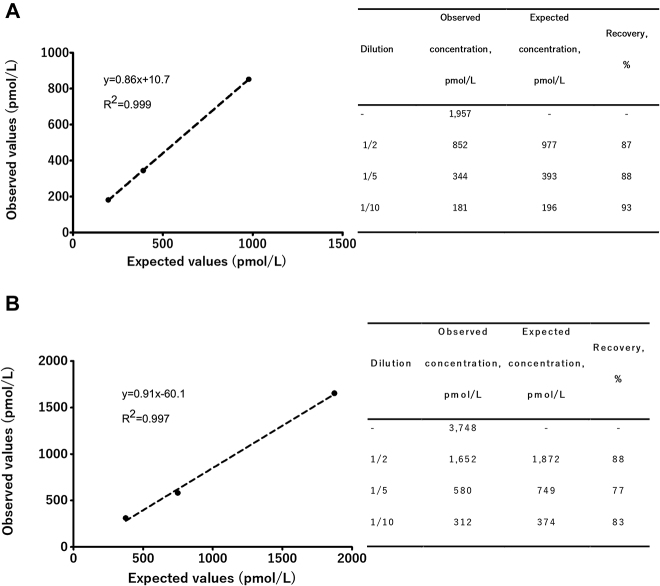
Atellica IM 1600 eE2 assay linearity studies starting from a serum sample with eE2 concentration of 1,957 pmol/L (A) and 3,748 pmol/L (B).

Correlation between measured and expected values was high (*R* ^2^ > 0.99), indicating good linearity for both sets of serial E2 dilutions. The average percent recovery for dilution series A and B was 89% and 83%, respectively, (Figure 2).

### Method comparison

**Figure 3:**
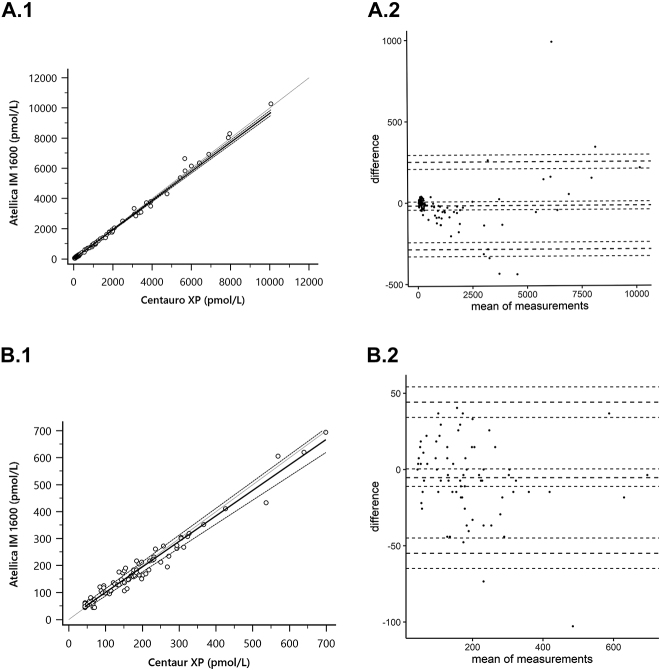
Method comparison. Passing–Bablok regression and Bland–Altman analyses comparing the Atellica IM 1600 eE2 and Advia Centaur XP eE2 assays. (A.1) E2 whole range concentration Passing–Bablok regression (n = 119). (A.2) E2 whole range concentration Bland–Altman plot (n = 119). (B.1) E2 concentrations <734 pmol/L Passing–Bablok regression (n = 76). (B.2) E2 concentrations <734 pmol/L Bland–Altman plot (n = 76).

Comparison of results obtained for serum samples using Atellica IM 1600 and ADVIA Centaur XP is shown in Figure 3. The Atellica IM 1600 assay demonstrated good correlation with ADVIA Centaur XP assay over the entire range (*R* ^2^ = 0.99) and at E2 concentrations <734 pmol/L (*R* ^2^ = 0.96). Passing–Bablok regression equation was as follows: for Atellica, it was 0.96 (95% CI: 0.95–0.98) × Centaur + 2.8 (95% CI: −4.9 to 8.7) for the entire range and 0.95 (95% CI: 0.89–0.99) × Centaur + 5.9 (95% CI: −2.76 to 14) for concentrations lower than 734 pmol/L. Bias between both methods was 18 pmol/L (95% CI: −43 to 6.9) for the entire range and −5.3 pmol/L (95% CI: −11 to 0.41) for concentrations lower than 734 pmol/L.

Comparison of results obtained for serum samples using Atellica IM 1600 and DRG Sensitive ELISA is shown in Table 3. The concentration of four serum samples analyzed using the DRG Sensitive ELISA was <11 pmol/L, which also presented undetectable results when analyzed using the Atellica IM 1600 (<43.3 pmol/L). The concentrations of 11 of 16 serum samples analyzed using the Sensitive ELISA were between 11.0 and 43.3 pmol/L, which presented undetectable results (<43.3 pmol/L) when analyzed using the Atellica IM 1600. Conversely, the concentration of eight of the nine serum samples analyzed using the DRG Sensitive ELISA was >43.3 pmol/L, which also presented detectable results when analyzed using the Atellica IM 1600.

**Table 3:** Low E2 serum samples comparison between DRG Sensitive ELISA and Atellica IM 1600 eE2 assays.

DRG Sensitive ELISA, pmol/L	n	Atellica IM 1600, pmol/L	n	Agreement, %
<11	4	<43	4	100%
11–43	16	≤43	11	69%
		>43	5	
≥43	9	≥43	8	89%
		<43	1	

n, number of measurements.

## Discussion

In this study, we assessed the analytical performance of the Atellica IM eE2 assay. Direct E2 immunoassays offer many advantages that include their commercial availability as automated assays, avoiding of time-consuming sample extraction, high throughput, and fast turnaround times. A method capable of quantifying across a wide dynamic range is an essential tool in clinical diagnostic laboratories, especially those engaged in fertility testing.

The assay performed well overall, demonstrating good linearity and imprecision across a typical clinical range. In this regard, according to Stanczyk et al. [2], we focused the evaluation of analytical imprecision and accuracy on E2 values <3,671 pmol/L. The intraday imprecision (CV from 1.8% to 4.0%) and interday imprecision (CV from 1.0% to 6.6%) were satisfactory at the five different concentrations of quality controls and serum pools and met the desirable specification for imprecision based on biological variation (<11%) [12]. Imprecision results are in agreement with those reported by the manufacturer and comparable with those previously reported for the eE2 assay in the ADVIA Centaur [14]. By means of reference certified materials, accuracy of the assay was evaluated at concentrations of 114, 690 and 1,340 pmol/L. The results obtained by the Atellica IM eE2 assay were compared with the certified values determined by the use of an isotope dilution gas chromatography mass spectrometry (GC–MS) reference method. Accuracy was good (<6.9%) and met the quality requirements of desirable specification for estradiol inaccuracy based on biological variation specifications (<8.3%) [12] at the values of 690 and 1,340 pmol/L. However, accuracy at the low level (114 pmol/L) was 28% and did not meet this requirement, which could be in agreement with the difficulties of direct assays to reliably evaluate low levels of E2 due to cross-reactivity with other steroid molecules or interferents not removed from the serum, which can result in significant bias [6], [7]. In fact, Ketha et al. [13] reported that analysis of data reported in a survey conducted in the College of American Pathologists showed that an E2 concentration of 106 pmol/L measured by LC–MS/MS (Liquid chromatography-tandem mass spectrometry) was overestimated (40% to 300%) by all the evaluated immunoassays platforms. In this survey, different ADVIA Centaur platforms presented an accuracy of approximately 40%.

Repeated measuring of an E2-free sample (eE2 assay diluent, n = 10) did not result in any detectable result. However, determination of the LOD was not possible as the commercial assay does not report values below the company-stated LOD (43 pmol/L). Instead, we were able to evaluate the limit of quantification as interassay CV of decreasing serum levels, obtaining a CV <20% at 53 pmol/L and a CV <10% at 125 pmol/L. This indicates that, in contrast with the difficulty of the assay in achieving good accuracy at low levels, imprecision remains very low in the entire range of the assay, also including low levels. This is also in agreement with results obtained with the ADVIA Centaur eE2 assay by Chen et al. [14].

Overall, results indicate that the method is reliable and appropriate for routine measurements of E2 concentrations beyond 43 pmol/L. However, as with other current direct immunoassays, the assay is not useful when evaluating very low serum E2 levels such as those expected in postmenopausal women receiving aromatase inhibitors for the treatment of breast cancer. In this scenario, methods need to be able to distinguish between suppressed levels of less than 3.7 pmol/L and pretreatment levels that commonly range from 37 to 55 pmol/L [3]. Mass spectrometry and indirect radioimmunoassays are currently the proposed alternatives to achieve this analytical sensitivity. Further improvement of direct immunoassays and E2 standardization is needed to better quantify low serum E2 levels in specific clinical situations.

Sample comparison between Atellica and ADVIA Centaur XP eE2 assays was performed covering all ranges of concentration evaluated and at concentration <734 pmol/L, which is the most common result obtained in our daily routine. No significant bias was observed both at the entire range and at concentration <734 pmol/L, and a high agreement between assays was noted. Finally, Atellica and the DRG E2 sensitive assays also presented good agreement and most of the samples below the LOD of Atellica (<43 pmol/L) were also <43 pmol/L by DRG.

In summary, the Atellica IM eE2 assay demonstrated acceptable imprecision and accuracy above the LOD of the assay and showed good agreement with the ADVIA Centaur XP.

## References

[1] Smy L, Straseski JA (2018). Measuring estrogens in women, men, and children: recent advances 2012–2017. Clin Biochem.

[2] Stanczyk FZ, Clarke NJ (2014). Measurement of estradiol—challenges ahead. J Clin Endocrinol Metab.

[3] Rosner W, Hankinson SE, Sluss PM, Vesper HW, Wierman ME (2013). Challenges to the measurement of estradiol: an endocrine society position statement. J Clin Endocrinol Metab.

[4] Reinsberg J, Bätz O, Bertsch T, Bewarder N, Deschner W, Drescher V (2009). Precision and long-term stability of different estradiol immunoassays assessed in a multi-center quality control study. Clin Lab.

[5] Vesper HW, Botelho JC, Vidal ML, Rahmani Y, Thienpont LM, Caudill SP (2014). High variability in serum estradiol measurements in men and women. Steroids.

[6] Yang DT, Owen WE, Ramsay CS, Xie H, Roberts WL (2004). Performance characteristics of eight estradiol immunoassays. Am J Clin Pathol.

[7] Coucke W, Devleeschouwer N, Libeer J-C, Schiettecatte J, Martin M, Smitz J (2007). Accuracy and reproducibility of automated estradiol-17beta and progesterone assays using native serum samples: results obtained in the Belgian external assessment scheme. Hum Reprod.

[8] Santen RJ, Demers L, Ohorodnik S, Settlage J, Langecker P, Blanchett D (2007). Superiority of gas chromatography/tandem mass spectrometry assay (GC/MS/MS) for estradiol for monitoring of aromatase inhibitor therapy. Steroids.

[9] Stanczyk FZ, Jurow J, Hsing AW (2010). Limitations of direct immunoassays for measuring circulating estradiol levels in postmenopausal women and men in epidemiologic studies. Cancer Epidemiol Biomarkers Prev.

[10] Stanczyk FZ, Lee JS, Santen RJ (2007). Standardization of steroid hormone assays: why, how, and when?. Cancer Epidemiol Biomarkers Prev.

[11] Stanczyk FZ, Clarke NJ (2010). Advantages and challenges of mass spectrometry assays for steroid hormones. J Steroid Biochem Mol Biol.

[12] Minchinela J, Ricós C, Perich C, Fernández-Calle P, Álvarez V, Domenech M (2014). Biological variation database, and quality specifications for imprecision, bias and total error (desirable and minimum).

[13] Ketha H, Girtman A, Singh RJ (2015). Estradiol assays – the path ahead. Steroids.

[14] Chen Y, Kinney L, Soldin SJ (2012). Performance evaluation of Siemens ADVIA Centaur® enhanced estradiol assay and a split sample comparison with liquid chromatography-tandem mass spectrometry. Clin Biochem.

